# Bone Marrow Transplantation Alters the Tremor Phenotype in the Murine Model of Globoid-Cell Leukodystrophy

**DOI:** 10.3390/jcm1010001

**Published:** 2012-01-19

**Authors:** Adarsh S. Reddy, David F. Wozniak, Nuri B. Farber, Joshua T. Dearborn, Stephen C. Fowler, Mark S. Sands

**Affiliations:** 1 Department of Internal Medicine, Campus box 8007, 660 South Euclid Avenue, Washington University School of Medicine, St. Louis, MO 63110, USA; E-Mail: adarsh.reddy@wustl.edu; 2 Department of Psychiatry, Campus Box 8134, 660 S. Euclid Avenue, Washington University School of Medicine, St. Louis, MO 63110, USA; E-Mails: wozniakd@psychiatry.wustl.edu (D.F.W.); farbern@psychiatry.wustl.edu (N.B.F.); dearborj@psychiatry.wustl.edu (J.T.D.); 3 Department of Pharmacology and Toxicology, University of Kansas, Lawrence, KS 66045, USA

**Keywords:** bone marrow transplantation, lysosomal storage disease, tremor, galactosylceramidase, globoid-cell leukodystrophy

## Abstract

Tremor is a prominent phenotype of the twitcher mouse, an authentic genetic model of Globoid-Cell Leukodystrophy (GLD, Krabbe’s disease). In the current study, the tremor was quantified using a force-plate actometer designed to accommodate low-weight mice. The actometer records the force oscillations caused by a mouse’s movements, and the rhythmic structure of the force variations can be revealed. Results showed that twitcher mice had significantly increased power across a broad band of higher frequencies compared to wildtype mice. Bone marrow transplantation (BMT), the only available therapy for GLD, worsened the tremor in the twitcher mice and induced a measureable alteration of movement phenotype in the wildtype mice. These data highlight the damaging effects of conditioning radiation and BMT in the neonatal period. The behavioral methodology used herein provides a quantitative approach for assessing the efficacy of potential therapeutic interventions for Krabbe’s disease.

## 1. Introduction

Globoid-cell leukodystrophy (GLD; Krabbe’s disease) is a rapidly progressive demyelinating disease caused by the deficiency of the lysosomal enzyme Galactosylceramidase (GALC) [1]. The disease is characterized by prominent CNS symptoms including delayed development, spasticity, and seizures. In humans, the age of onset of symptoms is usually around 6 months, with inevitable progression and death by around 2-3 years of age. Currently, the only available treatment in humans is bone marrow transplantation (BMT). Although BMT can be relatively effective if initiated in pre-symptomatic patients, it is not a cure and treated children continue to develop clinical signs. One important question is: are the continually progressing clinical signs due to GALC deficiency or are they treatment-related?

The murine model used to study GLD is the twitcher mouse which is deficient in the same enzyme as that of humans [2,3]. The eponymous and characteristic feature of the murine model is severe twitching or tremor. Other phenotypic features include decreased body weight, hindlimb ataxia, decreased mobility and a shortened lifespan. Similar to the human disease, the neuropathology consists of demyelination, axonal damage [4,5] with prominent inflammation, and the accumulation of lipid-laden macrophages (globoid-cells) throughout the CNS and the PNS. Although tremor is a prominent phenotype in the twitcher mouse, it has not been thoroughly characterized. Also, little is known about how the tremor is altered after various treatments, especially BMT following harsh conditioning regimens. In the current study, a detailed phenotypic characterization of the tremor in the twitcher mouse was performed using a force-plate actometer [6] that allowed recording of the movement-related force generated by small (7–13 g) mice. The power spectra and the locomotor activity of the twitcher mice were compared to the wildtype mice to reveal several important differences. The study also evaluated the effect of BMT on tremor and locomotor activity in both the twitcher and wild type mice. The results demonstrate that BMT and the harsh conditioning regimens associated with this procedure can superimpose additional clinical defects on this already devastating disease. 

## 2. Methods

### 2.1. Colony Maintenance

*Galc +/*–and wildtype mice on the C57BL/6J background were obtained from The Jackson Laboratory (Bar Harbor, ME) and maintained under the supervision of M.S.S. at Washington University School of Medicine. The *galc-/-* mice were obtained by *galc+/*– X *galc+/*– matings. The *galc* genotype was determined by twitcher-specific PCR [7]. All animals were allowed *ad libitum* access to food and water, except during brief (12-min or less) behavioral recording sessions. All animal experiments were approved by Institutional Animal Care and Use Committee at Washington University School of Medicine. 

### 2.2. BMT and Harmaline Injections

Neonatal pups were genotyped on day 2 or 3 and BMT was performed on post natal day 3 or 4. The mice received 400 rads of total body irradiation from a ^137^Cs source. The animals received an intravenous injection of 10^6^ nucleated bone marrow cells from a sex-matched *galc+/+*, GFP (+) donor [8] via the superficial temporal vein [9].

Harmaline (1-methoxy-3, 4-dihydro-β-carboline, H1392, Sigma, St. Louis, MO) at a dose of 15 mg/kg was injected intraperitoneally 12 minutes before the start of tremor monitoring on post natal day 36. 

The nomenclature for the various treatment groups and the number of animals used in the study is as follows: (a) Wt- untreated wildtype (n = 28), (b) Twi- untreated mutant (twitcher; n = 34), (c) BmtWt- wildtype mice treated with BMT (n = 12), (d) BmtTwi- twitcher mice treated with BMT (n = 7), (e) WtH- wildtype mice treated with harmaline (n = 11), (f) TwiH- twitcher mice treated with harmaline (n = 8), (g) BmtWtH- BmtWt mice treated with harmaline (n = 11), and (h) BmtTwiH- BmtTwi mice treated with harmaline (n = 7). 

### 2.3. Bone Marrow Engraftment

Since the current study was an extension of the previous study (Reddy *et al.*, 2011), and there was an overlap in the animals that were used in the abovementioned study with the current study. The engraftment levels from the various groups are described in results. 

### 2.4. Force Plate Actometer

The design of the force plate actometer and the principles used in the design were described previously [6]. For the current study a force-plate actometer was custom made to accommodate the relatively low body weight and impaired force production capabilities of the untreated twitcher mice. The mean weight of the twitcher mice at 36 days was 10.4 ± 1.6 grams compared to 16.9 ± 1.8 grams for wildtype mice at this age. The custom-made actometer used a carbon fiber/nomex composite material for the load plate, which weighed 57 g, was 3.2 mm thick and measured 24 cm × 24 cm. The sensing area was 20 cm × 20 cm, and the cage that confined the mouse to the load plate was constructed of 6.4 mm-thick clear polycarbonate with inside dimensions of 20 cm long by 20 cm wide by 15 cm high. A removable clear polycarbonate top was perforated with ventilation holes. The load plate was supported by four Model 31a miniature strain gauge load cells purchased from Honeywell/Sensotec (Columbus, Ohio). The load cells were calibrated to yield a force resolution of 0.2 gram-force. 

### 2.5. Actometer Data Acquisition and Analysis

The animals were acclimated for at least 30 minutes in the same room prior to tremor monitoring. Data recording was conducted between 2 pm and 6 pm. For mice given harmaline, the drug or saline was injected 12 minutes prior to recording. Data were collected for 6 min, but only the first minute, when movement was maximal, was used for the tremor analyses. The recordings from the transducers were collected at 100 samples/s. The 12-bit integer raw data files were acquired with a LabMaster interface (Scientific Solutions, Mentor, Ohio) that was controlled by a DOS-based Free Pascal program (http://www.freepascal.org). The data from the raw integer files were converted to text files and formatted by Free Pascal programs for further processing by commercially available software (see Statistics section below). Custom written Free Pascal programs were used to calculate distance traveled and the number of low mobility bouts (see below). 

**Figure 1:**
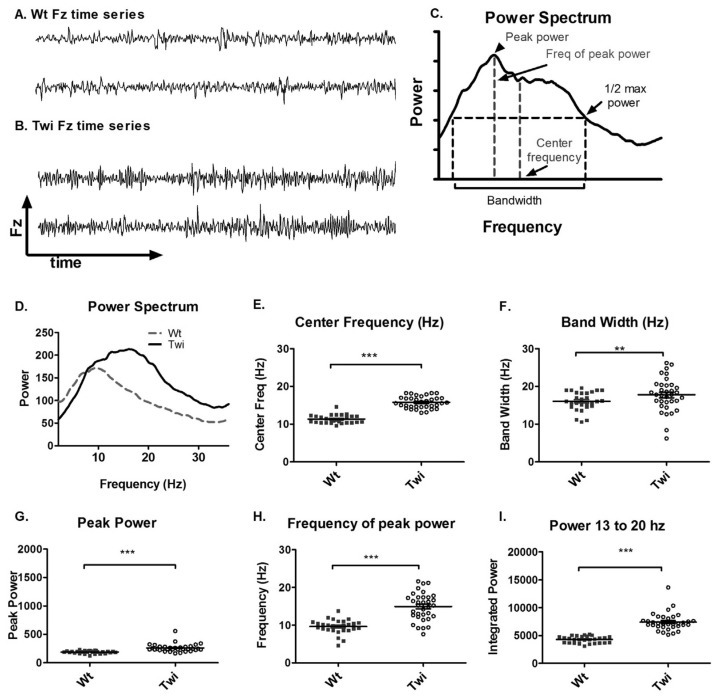
**Characterization of tremor in the Twi mice.** Representative Fz time series recording from a Wt mouse **(A)** and a Twi mouse **(B)** over 12 seconds (each series is 6 s). The X-axis represents time and the Y-axis represents force the vertical force variation (Fz) recorded by the force plate actometer. A Fourier transformation performed on the Fz time series data yields a power spectrum that shows how much power (variance) the Fz variation contains at each frequency of oscillation. The power spectrum can then be further analyzed to yield peak power, frequency at peak power, center frequency, and bandwidth. These data are represented diagrammatically on a hypothetical power spectrum plot **(C)**. The averaged power spectrum **(D)** of the Twi mice (solid black line) was shifted towards higher frequencies compared to the Wt mice (dashed gray line). The center frequency **(E)** was significantly increased in the Twi (open circles) compared to the Wt mice (filled squares). There was a significant increase in the band width **(F)** in the Twi compared to the Wt group. The peak power **(G)**, frequency at peak power **(H)** and the power between 13 and 20 Hz (I) were significantly increased in the Twi group compared to the Wt group. The horizontal bars represent the mean and the error bars represent the SEM (***p < 0.001, **p < 0.01).

The following data were extracted from the raw data files [6]: (a) **Fz**- the net force exerted by the animal at a particular 0.01-s “instant” was calculated as the sum forces on each of the four transducers that supported the load plate. The digitized Fz data obtained at 100 samples/s for the first minute of the recording session were formatted into 12 consecutive 5.00-s time series. Importantly, for the tremor analyses the Fz time series data were expressed as a percent of each mouse’s body weight. This normalization made it possible to compare the power spectra across genetic and treatment conditions without potential confounding by the body weight differences. Each time series (Fz(t)) was Fourier transformed using the fft function in MATLAB (The Mathworks, Inc., Natick, MA). A 500-point Hanning time-domain data window was used. The resulting 12 power spectra were averaged together to yield a single power spectrum for each mouse. The individual frequencies obtained after Fourier transformation were plotted as a continuous function (**power spectrum;** see Figure 1C) after filtering to retain frequencies between 2.5 and 30.0 Hz. (b) The **bandwidth** was defined as the difference between the upper and lower limit of the frequencies where the power was half that of the maximum. (c) The **center frequency** was calculated as the frequency co-ordinate of the vertical line bisecting the bandwidth. (d) The **frequency at peak power** was taken as the frequency at which the power was at its maximum. (e) **Power between 13 and 20 Hz** was obtained by integrating the area under the power spectrum curve between 13 and 20 Hz. The aforementioned power spectrum variables (a–e) were computed for each individual mouse, and these variables were then subjected to standard statistical treatments (see below). 

Although the tremor analyses were performed for the first minute of force-plate recordings, the variables for tracking and quantifying the mouse’s horizontal movements in the actometer were based on the entire 6-min session. (f) The **X-Y position** of the mouse on the force plate and (g) the **distance traveled** by the animal was calculated using the principle of moments and by calculating distance (in mm) between centers of force locations at successive time points 0.5 s apart, respectively [6]. The X-Y locations of the animal at various time points were plotted as a function of time to obtain the (h) **trajectory** of animal movement. (i) X-Y coordinates of the center of force as a function of time were additionally used to identify a **low mobility bout,** which was defined in terms of a virtual circle with a radius of 15.0 mm that was centered on the mouse as it moved across the load plate. When 5.00 s elapsed without movement beyond the perimeter of the circle, a low mobility bout was tallied, and the 5.00-s time interval was reset to 0 in order to “look for” the next bout. This measure gives an indication of a mouse’s proclivity to “stay in one place” regardless of the location of that place on the load plate.

### 2.6. Statistical Analyses

Systat (Systat Software Inc., Chicago, IL, USA) and Graphpad prism (Graphpad Software, Inc., La Jolla, CA) were used for generating graphs and performing statistical analyses. ANOVA or Kruskal-Wallis tests were used to compare different groups, and *post-hoc* multiple comparisons were done using Bonferroni tests.

## 3. Results

### 3.1. Tremor and Locomotion in Twitcher Mice

The qualitative differences in the Fz time series between the Wt and Twi mice are shown in Figure 1A and B. A Fourier transform was applied to the Fz time series to obtain a power spectrum (Figure 1C). The power spectra of different animals were further analyzed to obtain the peak power, frequency of peak power, center frequency, and bandwidth (Figure 1C). The averaged power spectra (Figure 1D) showed that the Twi mice had substantially higher power at higher frequencies compared to the Wt mice. The center frequency and the bandwidth in the Twi group were significantly higher than these variables for the Wt group (Figure 1E and F). The peak power (Figure 1G), frequency of peak power (Figure 1H), and the integrated power between 13 and 20 Hz (Figure 1I) were significantly increased in the Twi group compared to the Wt group (p < 0.001 for all three comparisons; t-test). The data for the Wt group are indicative of normal movements without any visible tremor, whereas the Twi mice exhibited the obvious tremor for which they are named. This analysis shows that the tremor of the twitcher mice manifests itself as relatively broad band, high-frequency force oscillations while on the force-plate.

**Figure 2:**
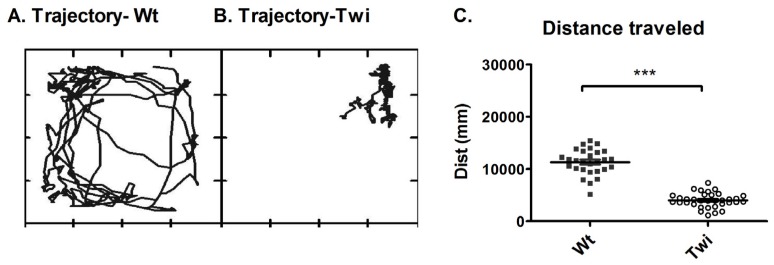
**Characterization of locomotor activity in the twitcher mouse.** The movement trajectory of representative Wt and Twi mice are shown in **(A)** and **(B)**, respectively. Each panel represents the movement of the mouse for duration of 1 minute. The box in which each movement trajectory is plotted represents the inside wall of the 20 cm × 20 cm cage that confined the mouse to the load plate. Each point in the panel represents the XY location of the mouse at a certain point of time. The total distance traveled during 6 minutes is shown in **(C)**. The total distance traveled by the Wt mice (filled squares) is significantly higher than that of the Twi mice (open circles). The horizontal bars represent the mean and the error bars represent the SEM (***p < 0.001).

The Twi group had decreased locomotion compared to that of the Wt group. Representative trajectories of these two groups are shown in Figure 2A and B. The total distance traveled was significantly lower in the Twi group compared to the Wt group (p < 0.001, t-test; Figure 2C).

**Figure 3:**
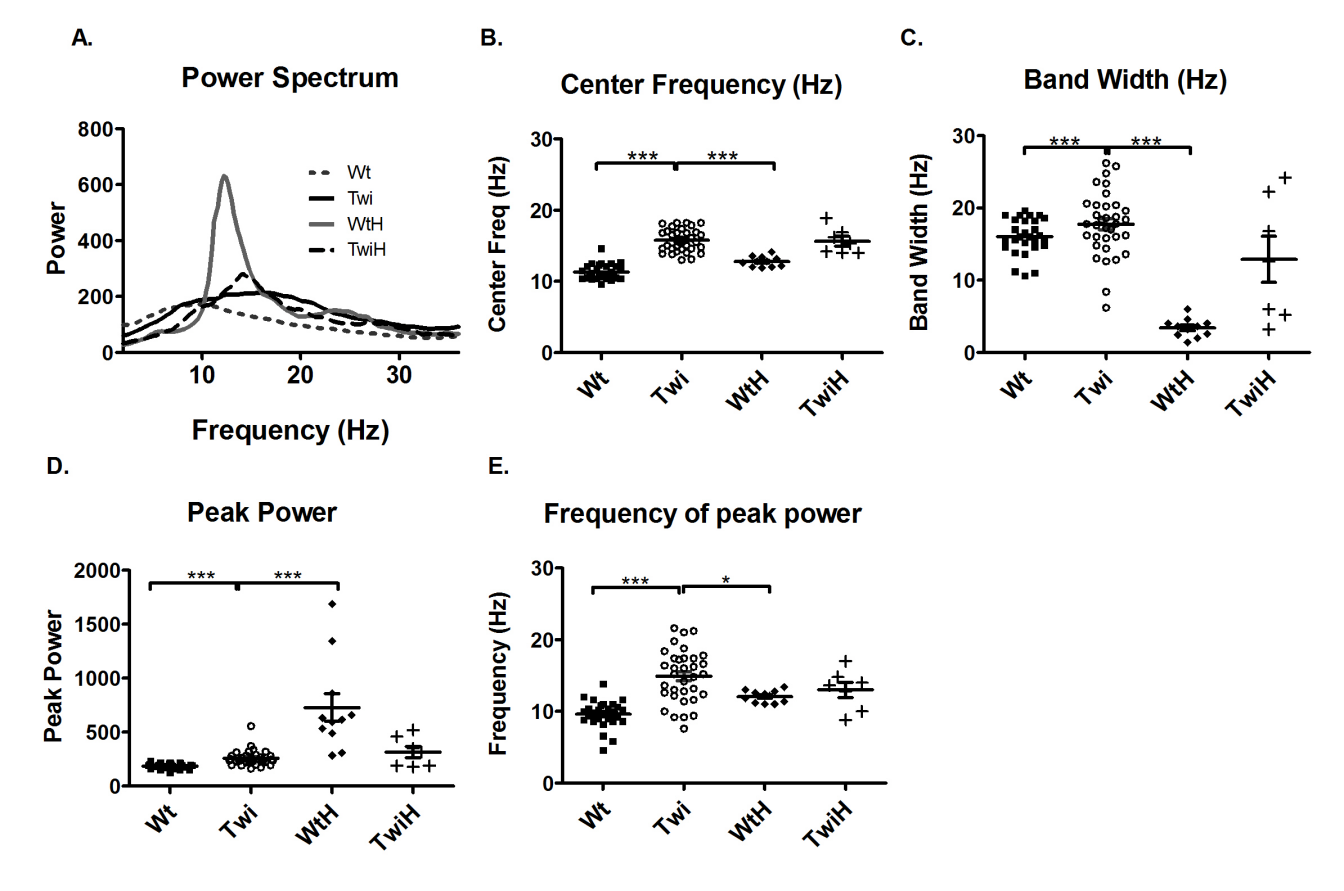
**Comparison of the tremor of the Twi mice with that of the tremor induced by harmaline.** The average power spectrum **(A)** of the Twi mice (solid black line) reflected a broadband tremor and that of the WtH mice (solid gray line) showed a characteristic narrow band 12 Hz tremor. The response to harmaline was blunted in the TwiH mice (dashed black line). The center frequency **(B)** and bandwidth **(C)** were significantly increased in the Twi group (open circles) compared to the WtH group (filled diamonds), which exhibited the expected characteristic narrow band tremor typically induced by harmaline in mice. The peak power **(D)** was significantly increased in the WtH group compared to the Twi group. The frequency at peak power was significantly decreased in the WtH group compared to the Twi group. There was no significant difference in the center frequency, bandwidth, peak power and the frequency of peak power between the Twi and TwiH groups (plus symbols). Horizontal bars represent the mean and the error bars represent the SEM (***p < 0.001, **p < 0.01 and *p < 0.05).

One way to validate the tremor recorded by this newly developed actometer is by comparing it to a relatively well accepted pharmacological tremor model (harmaline treatment) [10]. Therefore, the tremor in the Twi mouse was compared to the tremor in the wildtype mice injected with harmaline (WtH). Harmaline induces a characteristic narrow-band, near 12-Hz tremor. Comparison of the Twi and the WtH animals revealed a robust between-group difference in the averaged power spectra (Figure 3A). As quantified in terms of the mean of the center frequencies, the Twi mice (15.8 Hz) had significantly higher values than the WtH group (12.2 Hz) (p < 0.001; Bonferroni test, Figure 3B). The WtH group had a near-12.2 Hz narrow peak, and the bandwidth was significantly less than that of the Twi group (p < 0.001; Bonferroni test, Figure 3C). There was no statistically significant difference between the distance traveled and the number of low mobility bouts between the WtH and the Twigroups (data not shown), probably because tremor-inducing doses of harmaline in intact animals suppresses locomotion [10]. 

Interestingly, when twitcher mice were injected with harmaline (TwiH), there was a blunted response with no statistically significant change in center frequency or bandwidth compared to the Twi group (Figure 3B and C). In the WtH group, the peak power was significantly higher (p < 0.001; Bonferroni test; Figure 3D) while frequency of peak power was significantly lower (p < 0.05; Bonferroni test; Figure 3E) compared to the Twi group. There was no significant difference between the TwiH and WtH groups with respect to the distance traveled or the number of low mobility bouts (data not shown). 

### 3.2. Effect of Treatment on Tremor

In order to directly assess the effect of myeloreductive conditioning and BMT on the power spectra, tremor quantification was performed on both wildtype and twitcher mice that received BMT (BmtWt versus BmtTwi). The engraftment in various groups is essentially as reported in the study by Reddy *et al.*, 2011. BmtWt group had a mean +/– standard error engraftment of 20.52 +/– 3.077, BmtTwi group was at 12.1 +/– 1.9 and the AAVBMT group was 16.09 +/– 4.0. The engraftment level in the untransplanted animals was less than 1%. 

**Figure 4:**
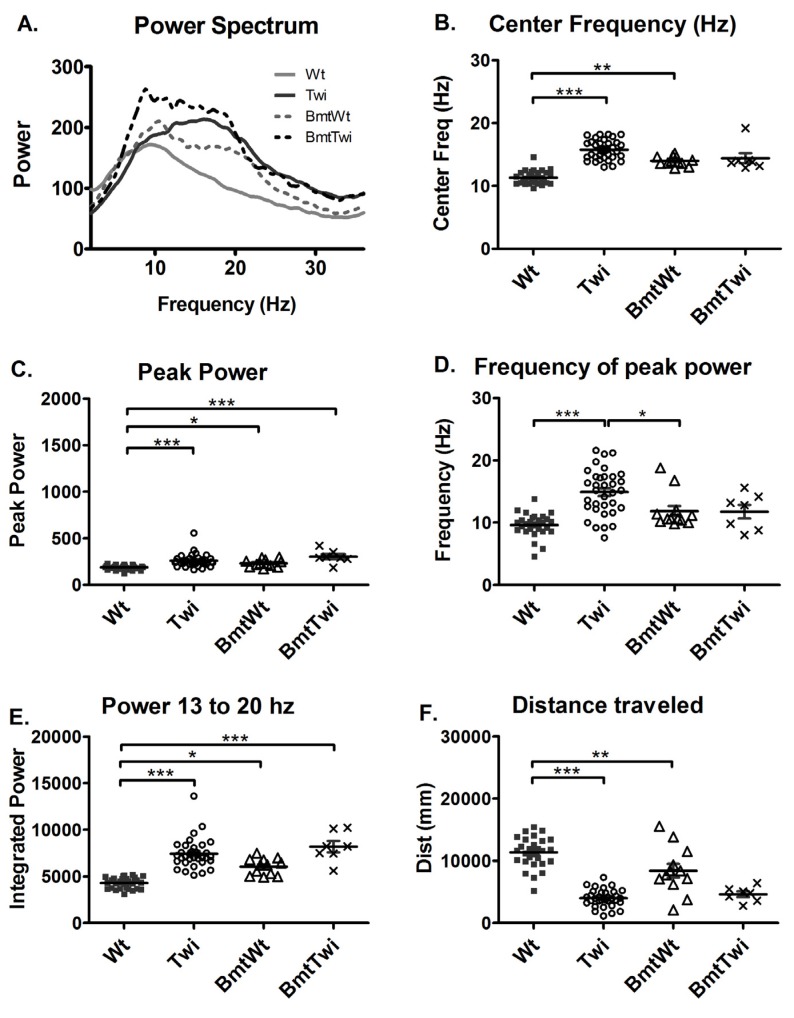
**Effect of BMT on power spectra.** The averaged power spectrum **(A)** of the BmtWt group (dashed gray line) is shifted upward and rightward compared to the Wt group (solid gray line). Similarly, the averaged power spectrum in the BmtTwi group (dashed black line) is shifted upward across a broad frequency band compared to the Twi group (solid black line). The center frequency **(B)** was significantly increased in the BmtWt group (open triangles) compared to the Wt group (filled squares). There was no significant difference between the untreated mut (open circles) and BmtTwi groups (cross marks) in the center frequency. The peak power **(C)**, frequency of peak power **(D)** and the power between 13 and 20 Hz **(E)** was significantly increased in the BmtWt group compared to the Wt group. Compared to the Twi group, the BmtTwi group showed no significant difference in the center frequency **(B)**, peak power **(C)**, frequency of peak power **(D)** and power between 13 and 20 Hz **(E)**. The distance traveled by the BmtWt group was significantly decreased compared to the Wt group **(F)**. The horizontal bars represent the mean and the error bars represent SEM (***p < 0.001, **p < 0.01 and *p < 0.05).

The BmtWt group had greater power in both the frequencies around 10–12 Hz and in the higher frequencies in the 13–20 Hz range compared to the Wt group (Figure 4A). The BmtTwi group had greater power in the near 10 Hz lower frequency range compared to the Twi group. The center frequency of the BmtWt group was significantly increased compared to the Wt group (p < 0.01; Bonferroni test; Figure 4B). The bandwidth variable (data not shown) did not indicate any significant between-group effects. The peak power (Figure 4C) and the power between 13 and 20 Hz (Figure 4E) was significantly increased in the BmtWt group compared to the Wt group but not in the BmtTwi group compared to the Twi group. The frequency of peak power was not significantly different in the BmtWt group compared to the Wt group (Figure 4D). There was no significant difference in the frequency of peak power between the BmtTwi group and the Twi group (Figure 4D). The total distance traveled was significantly decreased (decrement in normal function) in the BmtWt group compared to the Wt group (Figure 4F). 

### 3.3. Harmaline Response in BMT Animals

**Figure 5:**
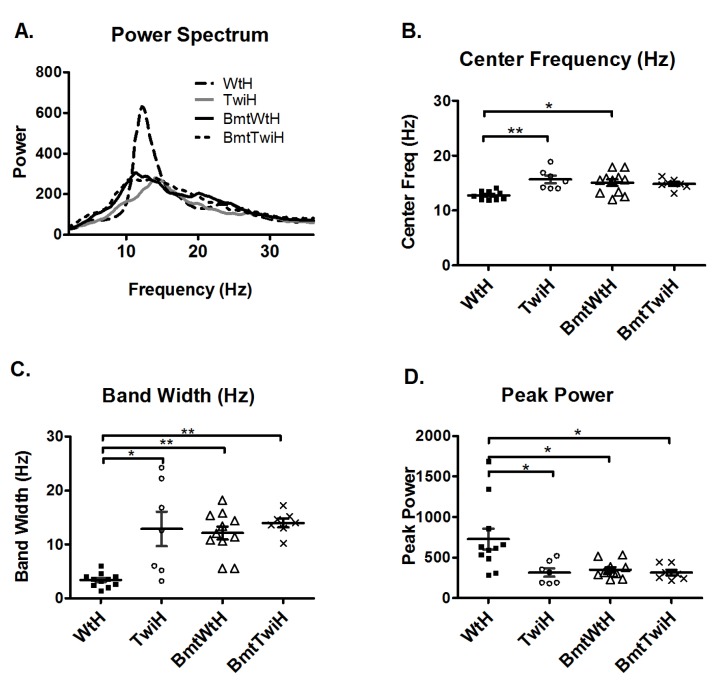
**Harmaline tremor response in the BMT-treated animals.** Averaged power spectra of various groups treated with harmaline **(A)**. The averaged power spectrum of WtH group (solid gray line) appears different compared to the averaged power spectrum of the other groups in **(A)**. The averaged power spectra of the BmtWtH (solid black line), TwiH (dashed black line), and BmtTwiH (solid gray line) appear very similar to each other. The center frequency **(B)** and the bandwidth **(C)** were significantly increased in the BmtWtH group (open triangles) compared to the WtH group (filled squares). The peak power **(D)** was significantly decreased in the BmtWtH group compared to the WtH group. There was no significant difference in center frequency, bandwidth or peak power between the TwiH (open circles) and BmtTwiH groups (cross marks). Horizontal bars represent the mean and the error bars represent the SEM (***p < 0.001, **p < 0.01 and *p < 0.05).

The BMT-treated animals that had additional higher frequencies in the averaged power spectra were compared to mice that received harmaline after BMT (BmtWtH and BmtTwiH). Interestingly, the mice in the BmtWtH group were more resistant to harmaline-induced tremors than the WtH group (Figure 5A). The response to harmaline in the BmtTwiH group was similar to the TwiH group (Figure 5A). The center frequency was higher and the bandwidth was broader in the BmtWtH group compared to the WtH group (Figure 5B and C). The peak power was significantly lower in the BmtWtH group compared to the WtH group (Figure 5D). There was no significant difference in the center frequency, bandwidth and peak power between the TwiH and BmtTwiH groups (Figure 5B, C and D). There was no significant difference in the distance traveled or in the number of low mobility bouts in the groups compared in Figure 5 (data not shown). 

### 3.4. Cerebellar Pathology

**Figure 6:**
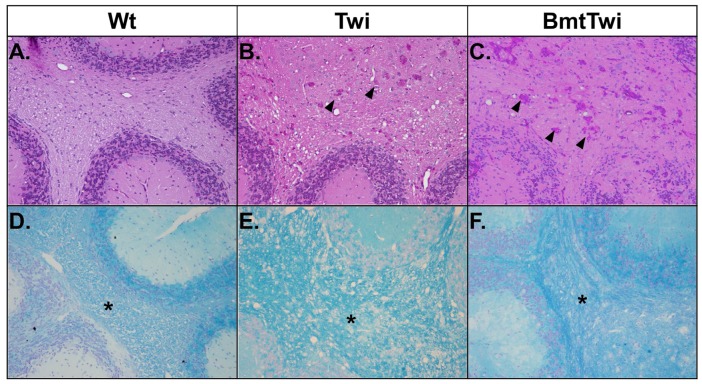
Histology- PAS and LFB staining. There are no identifiable PAS-positive cells in the cerebellum of the Wt animal **(A)**. In the Twi **(B)**, there are numerous pink multinucleated globoid cells (arrowheads). There is no obvious decrease in the number of PAS-positive cells in the BmtTwi group **(C)**. Comparison of LFB staining in the cerebellar white matter (asterisk in D–F) and cerebellar folia shows a disruption of myelin architecture (blue staining) in the Twi **(E)** mouse compared to the Wt **(D)** animal. In the BmtTwi group **(F)**, the myelin architecture remains disrupted, similar to the Twi group.

The brains of the various treatment groups were evaluated for the presence of globoid cells (multinucleated macrophages with storage material) and myelination using Periodic acid-Schiff (PAS) and Luxol fast blue (LFB) staining, respectively (Figure 6). When stained for PAS, the cerebellar white matter from a Wt mouse shows no globoid cells (Figure 6A), in contrast the brain from a Twi mouse contains a large number of multinucleate cells with pink staining cytoplasm (Figure 6B, arrowheads). There is no apparent decrease in the number of globoid cells in the BmtTwi mouse compared to the Twi mouse (Figure 6C). When the brains are stained for LFB, the Wt group shows uniform myelination in the cerebellar white matter (asterisk in Figure 6D). In contrast, the architecture of the myelin in the Twi group appears less well organized, probably secondary to inflammatory edema (Figure 6E). In the BmtTwi group, the myelin remains disorganized, similar to that observed in the Twi group (Figure 6F).

## 4. Discussion

In the current study, a detailed quantitative characterization of the tremor phenotype (movement-related force oscillations) in the twitcher mice was performed using a force-plate actometer. The tremor in the twitcher mouse was seen as increased power at relatively higher frequencies with a broader bandwidth than the movement-related force oscillations exhibited by the wildtype mice moving normally on the force plate. An incidental finding in these studies was that the stereotypical tremor produced by harmaline was significantly altered in the twitcher mice. Harmaline is believed to disrupt the olivocereballar circuits [11]. The altered response of twitcher mice to harmaline is consistent with the deficiency of GALC leading to inflammation and demyelination in the white matter tracts of the cerebellum [12,13] including the olivocerebellar nucleus. 

The validity of tremor monitoring in evaluating the effects of various therapies was also determined. Hematopoietic stem cell transplantation (either using umbilical cord blood or bone marrow) is the only available therapy for Krabbe’s disease in humans [14,15]. Bone marrow transplantation also prolongs the lifespan in the twitcher mouse [12,16,17]. However, the effect of radiation conditioning (in preparation for BMT) and transplantation of donor bone marrow cells on the prominent tremor phenotype exhibited by the twitcher mouse had not heretofore been determined. Twitcher mice receiving BMT had power spectra that had greater power in higher frequencies, implying a worsening of tremor. This is contradictory to what is observed in terms of other measures like lifespan seen in previous studies [12,13]. Interestingly, wildtype animals receiving only BMT also had altered power spectra and were resistant to the effects of harmaline compared to the wildtype animals that did not receive BMT. Bone marrow transplantation using myeloreductive conditioning (400 rads of total body irradiation) is known to cause cerebellar dysplasia in neonatal mice [18]. The blunted response of the harmaline-treated animals receiving BMT is also consistent with the conditioning regimen causing cerebellar damage, including the olivocerebellar nucleus. Conditioning and BMT could also be adversely affecting some as yet unknown regions in the brain or periphery to give the same abnormal response. The current study thus highlights a possible harmful effect of conditioning in treating the disease. These effects could be explained by the presence of rapidly proliferating cells in the cerebellum during the time of conditioning and BMT [19]. Although, the current study uses myeloreductive conditioning at post natal day 3 or 4, similar and perhaps more severe function-compromising effects could be expected in studies that use fully myeloablative regimens [16,17]. Although conditioning regimens used in newborn children [15] typically do not involve ionizing radiation, the myeloablative drugs are nonetheless highly toxic and may superimpose additional abnormal phenotypes on the already complex Krabbe’s disease presentation. This could further complicate the interpretation of the therapeutic benefits of BMT for GLD. 

This study establishes the use of a custom-built, ultra-sensitive force-plate actometer in evaluating the effects of various therapies for the twitcher mice. It also emphasizes the need to evaluate the impact of various therapeutic approaches on a wide variety of functions before drawing conclusions on their safety and efficacy. 
